# Care Staff Perspectives on Using Mobile Technology to Support Communication in Long-Term Care: Mixed Methods Study

**DOI:** 10.2196/21881

**Published:** 2020-09-29

**Authors:** Rozanne Wilson, Jeff Small

**Affiliations:** 1 School of Audiology and Speech Sciences Faculty of Medicine The University of British Columbia Vancouver, BC Canada

**Keywords:** mobile apps, mobile phone, caregivers, dementia, communication, patient care

## Abstract

**Background:**

Long-term care (LTC) homes provide 24-hour care for people living with complex care needs. LTC staff assist older adults living with chronic conditions such as Alzheimer disease, related dementias, and stroke, which can cause communication disorders. In addition to the complex cognitive challenges that can impact communication, further difficulties can arise from cultural-language differences between care staff and residents. Breakdowns in caregiver-resident communication can negatively impact the delivery of person-centered care. Recent advances in mobile technology, specifically mobile devices (tablets and smartphones) and their software apps, offer innovative solutions for supporting everyday communication between care staff and residents. To date, little is known about the care staff’s perspectives on the different ways that mobile technology could be used to support communication with residents.

**Objective:**

This study aims to identify care staff’s perspectives on the different ways of using devices and apps to support everyday communication with adults living in LTC homes and the priority care areas for using mobile technology to support communication with residents.

**Methods:**

This descriptive study employed concept mapping methods to explore care staff’s perspectives about ways of using mobile technology with residents and to identify the usefulness, practicality, and probable uses of mobile technology to support communication in priority care areas. Concept mapping is an integrated mixed methods approach (qualitative and quantitative) that uses a structured process to identify priority areas for planning and evaluation. In total, 13 care staff from a single LTC home participated in this study. Concept mapping includes 2 main data collection phases: (1) statement generations through brainstorming and (2) statement structuring through sorting and rating. Brainstorming took place in person in a group session, whereas sorting and rating occurred individually after the brainstorming session. Concept mapping data were analyzed using multidimensional scaling and cluster analysis to generate numerous interpretable data maps and displays.

**Results:**

Participants generated 67 unique statements during the brainstorming session. Following the sorting and rating of the statements, a concept map analysis was performed. In total, 5 clusters were identified: (1) *connect*, (2) *care management*, (3) *facilitate*, (4) *caregiving*, and (5) *overcoming* *barriers*. Although all 5 clusters were rated as useful, with a mean score of 4.1 to 4.5 (Likert: 1-5), the care staff rated cluster 2 (*care management*) as highest on usefulness, practicality, and probable use of mobile technology to support communication in LTC.

**Conclusions:**

This study provided insight into the viewpoints of care staff regarding the different ways mobile technology could be used to support caregiver-resident communication in LTC. Our findings suggest that care management, facilitating communication, and overcoming barriers are 3 priority target areas for implementing mobile health interventions to promote person-centered care and resident-centered care.

## Introduction

### Background and Rationale

By 2021, it is predicted that the number of adults aged 65 years or older will account for approximately 25% of the Canadian population [1,2]. Moreover, by 2030, the proportion of adults who are aged 85 years or older will account for approximately 20% of all older adults and 3% of the Canadian population. As the population ages, there will be an increase in the number of older adults living with multiple chronic health conditions that contribute to physical, functional, and cognitive decline, resulting in complex care needs that require the services offered in long-term care (LTC). Indeed, most LTC residents are aged older than 85 years, with 80% of residents being functionally dependent on care staff, whereas an estimated 90% of all residents are living with at least some cognitive impairments, including dementia [3].

In LTC homes, care staff are responsible for meeting the complex health care needs of residents. For instance, nursing staff administer medication and coordinate patient care, whereas resident care aides and/or personal support workers engage residents in basic activities of daily living (ADLs; eg, dressing, bathing, eating). The ability of care staff to support the complex needs of residents living with physical and functional limitations is further complicated when a communication disorder (eg, aphasia) or language differences are also present. Communication impairments are associated with various chronic conditions that are prevalent in LTC (eg, dementia, stroke), and breakdowns in communication during interpersonal and task-focused activities [4] can strain the relationship and lead to unmet care needs [5]. Furthermore, care staff–resident communication can be challenged by cultural-language barriers [6-8]. Efforts have been made to support people living with communication barriers in the LTC setting through the development and implementation of evidence-based communication strategies [9-12] and language translation supports [13]. However, these current solutions require training, staffing and time resources, and could be inaccessible when needed, making residents vulnerable to unmet needs and social isolation [14]. With some training, recent advances in mobile technology, which includes mobile devices (tablets/smartphones) and their software applications (apps), have the potential to yield innovative solutions for supporting care staff-resident communication and prevent or overcome communication barriers.

The increased sophistication of mobile technology has permitted the successful merging of multiple features (eg, portability, communication function, on-demand powerful computer technology, and a huge range of app options) into a single device that can be used to provide services that aim to improve health outcomes [15]. The health care industry has been driving the growth of mobile technology due to an increased demand for using technology to support health care practice and delivery, otherwise known as mobile health (mHealth) [16]. This demand has contributed to a large increase in the number of consumer mHealth apps available in the app marketplace [17] for various health categories (eg, diabetes, weight loss). These mHealth apps are designed to run on smartphone or tablet computer operating systems (eg, iOS and/or Android) to support a range of health care practices, including decision support aids, educational information, health monitoring, health promotion, staff-client communication, and care of the elderly [16]. mHealth apps are cost-effective, innovative point-of-care tools that immediately connect health care staff with information, presented in multiple forms (eg, text, images, sound, touch) and can be used to improve communication between health care staff and patients [18]. Although it is important to recognize the limitations of implementing this technology in health care and to ensure that mHealth apps meet a standard for quality and safety [19,20], mHealth apps continue to bring added value to health care practice and delivery, including accessibility, convenience, lower cost of health care delivery, and promotion of healthy choices [20]. Undoubtedly, the continued growth in mHealth will impact the use of this technology by both professionals [18,21,22] and health consumers [23,24].

Importantly, there is a demand for mHealth solutions to support the growing aging population, people living with chronic conditions, and patient-centered care [25-27]. In LTC, immediate access to information and the interactivity of mHealth interventions have the potential to support care staff–resident relationships as well as to improve the quality and quantity of resident care. Although it has been shown that certain health care providers (eg, physicians, nurses) have incorporated mHealth in their professional practice and during health care practice or delivery [18,28,29], there is a gap in our understanding of how LTC staff could adopt mobile technology in their daily care practice. As mobile technology offers many innovative apps for the care of older adults living in LTC homes [30], there is a need to better understand care staff utilization of currently available mainstream communication apps (cApps) to support residents during the completion of daily activities. This includes augmentative and alternative communication (AAC) apps designed for adults living with communication impairments (eg, *Proloquo2Go*) as well as translation apps (eg, *Google Translate*). The first step to better understand mHealth utilization in LTC is to examine the experiences and perspectives of care staff about the ways that mobile technology could be used to support care staff–resident communication as well as the priority care areas for using mobile technology to support communication. Ultimately, this knowledge could be used to facilitate meeting residents’ physical care and psychosocial needs.

### Research Aims

This study aimed to better understand LTC care staff perspectives on using mobile technology to support everyday communication with residents during activities of daily living. The study’s objectives were to identify the different ways that care staff would use mobile technology to support communication with residents living in LTC; the level of importance of the different ways of using mobile technology by examining their usefulness, practicality, and probable use; and priority care contexts for using mobile technology to support communication with residents.

## Methods

### Setting and Participants

Participants were recruited from a single LTC home in Vancouver, British Columbia, Canada. A purposive sample of full-time and part-time day and evening care staff who had direct interaction with residents during daily activities (eg, resident care aides, nurses) were included in this study. In addition, administrative staff and casual staff were invited to participate. The research team worked with a staff liaison to coordinate study information sessions for the morning and the evening shift on each care unit (ie, floor). A second information session was scheduled for care staff who were not able to attend the initial information session. A total of 36 care staff attended the information sessions. At the end of each information session, the care staff were asked to review the consent form and ask any questions they may have. All participants provided written consent before participation in this study. This study was approved by the University of British Columbia Research Ethics Board (H15-00270).

### Design, Data Collection, and Analysis

To better understand care staff’s perspectives on the use of mobile technology to support everyday communication with residents living in LTC homes, this study used concept mapping, also known as group concept mapping, to engage care staff in the research process. Concept mapping is a mixed methods approach that involves a structured process to integrate qualitative and quantitative data. Although historically used for program planning and evaluation [31], concept mapping has also been used for a wide range of studies, including measurement development [32-35], public health priority setting and program development [36,37], examining patient experience for quality improvement projects [38-40], understanding caregiver perspectives around care issues [41], and developing evidence-based public health care practices [42]. Concept mapping permits a diverse participant group of any size, in a wide range of settings, identifies participants’ perspectives, and visually represents their viewpoints about a focused topic on a map [43-45]. In addition, the visual display outputs derived from the concept mapping data show how topic ideas are related to each other and can reveal which ideas are more important, appropriate, or relevant [44]. A recent detailed description of this method can be found in a study by Trochim and Mclinden [45].

This study used 5 phases of the concept mapping method to identify the different ways that mobile technology could be used in LTC to support everyday communication and to better understand actionable areas to target the use of mobile technology during daily activities in LTC: (1) preparation—the development of the focused prompt; (2) idea generation—brainstorming and statement analysis or synthesis; (3) structuring—unstructured statement sorting, followed by rating statements; (4) representation—performing concept mapping analyses, including multidimensional scaling, hierarchical cluster analysis, and bivariate plots; and (5) interpretation—research group examines maps and agrees on the number of clusters as well as their names and descriptions. These phases are described below. Following the completion of phase 5, the final phase of concept mapping, phase 6 (ie, utilization), was undertaken and involved the reporting and dissemination of the research findings. Concept mapping was employed in this study for the following key reasons: (1) the approach uses a structured process that encourages a participatory method (ie, care staff engagement) to data collection and analysis; (2) the approach generates output that is more comprehensive than interviews [46](3) the method can be tailored to specific needs of the study by offering a level of flexibility to data collection (eg, both web-bas and face-to-face options). For example, having both web-based and face-to-face data collection options can increase the number of care staff participants (eg, casual staff, night staff) by offering a solution for overcoming scheduling and time constraints inherent to the work setting; and (4) the approach is efficient, requiring less time and research-intensive resources during the data collection and data analysis phases than traditional focus group interviews (eg, no transcription and coding involved).

#### Phases 1 and 2: Preparation and Statement Generation

To identify the different ways that mobile technology could be used to support caregiver-resident communication, a single focused prompt, “A specific way that mobile technology [eg, smartphones, tablets, and their applications (apps)], could be used to help everyday communication between residents and care staff during daily activities is...,” was used to generate statements. The statement generation step took place during 2 in-person group sessions. Participants were asked to independently write down their responses to the focused prompt and then share ideas as a group. Statements generated during the group discussion were recorded on a list visually available to all participants. The brainstorming activity ended after the participants indicated that all possible ideas were listed. Within 1 week of the brainstorming activity, the statements were consolidated by removing duplicates and overlapping or similar ideas. Next, participants were invited, via email, to individually complete the statement structuring phase.

#### Phase 3: Statement Structuring

To better understand target areas for using mobile technology to support communication in LTC, care staff completed statement sorting and ratings. For unstructured statement sorting, participants were asked to independently group the statements generated in phase 2 into piles based on how similar in meaning the statements were to one another. Care staff were instructed to categorize the statements in a way that *made sense* to them and to provide a name for each pile. The participants were also informed that each statement must be in a pile, that a statement can belong to only one pile, and that the creation of 10 to 20 piles is typical. Next, regardless of whether they had used mobile technology or not, care staff were asked to rate each statement in terms of 3 dimensions: (1) usefulness, or the degree to which using the app, as stated, would help or enhance everyday communication with residents during their care practice; (2) practicality, or how feasible would it be to use the app, as stated, to support everyday communication during their care practice; and (3) probable use, or how likely it would be that they would use the app, as stated, with residents to support everyday communication. All statements were rated on a 5-point Likert scale: *1=not at all*; *2=somewhat*; *3=moderately*; *4=very*; and *5=extremely*. Participants had the option to complete the sorting and rating steps on the web using Concept System Global MAX [47] or offline by sorting paper cards and rating sheets. The first author entered the data collected offline into Concept System Global MAX.

#### Phases 4 and 5: Representation and Interpretation

Once data from phase 3 were sorted and rated by participants, they underwent analysis to produce a series of concept maps. First, a multidimensional scaling analysis was used, whereby a point map and a point rating map were generated. The point map is relational, with separate points on the map corresponding to each statement with other statements. Points that are closer together indicate that sorters generally grouped these statements into piles. The point rating map represents an overlay of the point map and the average rating for each statement across participants [43]. Second, a hierarchical cluster analysis was conducted, which divides statements on the point map into clusters that represent conceptual groupings of the original set of statements [43]. From this analysis, point cluster maps were generated, which represents the overlap between the point map and the cluster analysis. Cluster maps provide an *overall picture* that represents the content of the concept being studied. In this instance, the different ways that mobile apps could be used to support everyday communication with residents living in LTC homes. Of note, cluster shape holds meaning, with wider clusters indicating a broader concept and a compact cluster representing a narrower concept [43]. Clusters that are closer to the middle of the map indicate that some statements within the cluster were also sorted with statements included in another cluster, representing a *bridging item*. During this step, the research team generated and reviewed several point cluster maps to determine the number of clusters that the statements should be grouped into (ie, final cluster solution). Research team consensus was used to decide the final cluster solution as well as to confirm the cluster names and descriptions. All subsequent analyses were based on the final cluster solution. Three cluster rating maps were generated, which represented the average participant ratings for each statement in a cluster, along the dimensions of usefulness, practicality, and probable use in practice. Clusters with higher values contained statements that received higher average ratings from the care staff participants.

Finally, Go-Zone analyses were performed, which visually display the relationship between 2 variables based on pairwise comparisons of cluster ratings: (1) usefulness and practicality, (2) usefulness and probable use, and (3) practicality and probable use. A Go-Zone analysis generates a bivariate graph that displays 4 quadrants that are divided based on the mean rating of each of the 2 variables. The upper right quadrant (quadrant 4) represents statements that are above average on both variables, thus indicating the *go-to care zone*, or priority (actionable) ways of using mobile apps to support everyday communication with residents living in LTC homes. Conversely, the bottom left area of the graph (quadrant 2) represents statements that are deemed lower on both variables, or the *no-go care zone* statements, or low priority ways of using mobile apps with residents. Following the analyses, based on the final cluster solution, the research team convened for phase 5 to interpret the findings. All concept mapping data analyses were conducted on the web using Concept System Global MAX.

## Results

### Participants

Of the care staff who attended the information sessions (n=36), 16 provided consent and 13 participated in this study. Of those who attended the information sessions but did not participate, 4 indicated that they were not interested during the information session and 16 were lost at follow-up. Although casual staff and administrative staff were invited to participate via a paper information package provided in their staff mailbox, none expressed an interest to participate. All participants completed at least one step (brainstorming: n=11; sorting: n=8; and rating: n=9; Table 1). Five participants completed the sorting and/or rating on the web at their convenience, and 4 participants completed these steps offline during an in-person meeting with the first author (RW). Overall, the majority of participants identified as female (12/13, 92%), ranging in age from 24 to 60 years (mean 45.4 years, SD 13.4), were residential care aides or health care aides (9/13, 69%), indicated English as their primary language (9/13, 69%), and spoke more than one language (9/13, 69%). Participants worked for an average of 12.7 years (SD 10; range 2-35) in the LTC setting. 

**Table 1:** Participant characteristics for each data collection step.

Characteristics		Overall^a^ (n=13)	Concept mapping steps		
			Brainstorming (n=11)	Sorting (n=8)	Rating (n=9)
**Age (years)**					
	Mean (SD)	45.4 (13.4)	43 (13.5)	45.9 (14.5)	45.2 (13.7)
	Range^a^	24-60	24-60	24-60	24-60
**Gender**					
	Female, n (%)	12 (92)	10 (91)	7 (88)	8 (89)
English primary language, n (%)		9 (69)	8 (73)	5 (63)	6 (67)
**Number of years working in long-term care**					
	Mean (SD)	12.7 (10)	12.5 (10.9)	11.2 (8.4)	10.7 (8.1)
	Range^b^	2-35	2-35	2-28	2-28
**Job title, n (%)**					
	Residential care aide or attendant	7 (54)	7 (64)	3 (38)	4 (44)
	Health care aide or assistant^a^	2 (15)	1 (9)	2 (25)	2 (22)
	Licensed practical nurse	1 (8)	1 (9)	1 (13)	1 (11)
	Activity therapist (eg, art, music)	2 (15)	2 (18)	1 (13)	1 (11)
	Dietician^a^	1 (8)	0 (0)	1 (13)	1 (11)

^a^One health care aide or assistant and the dietician did not participate in the in-person brainstorming step but did participate in the sorting and rating steps.

^b^One participant did not provide this information.

### Generated Statements and Cluster Map Analysis

**Figure 1:**
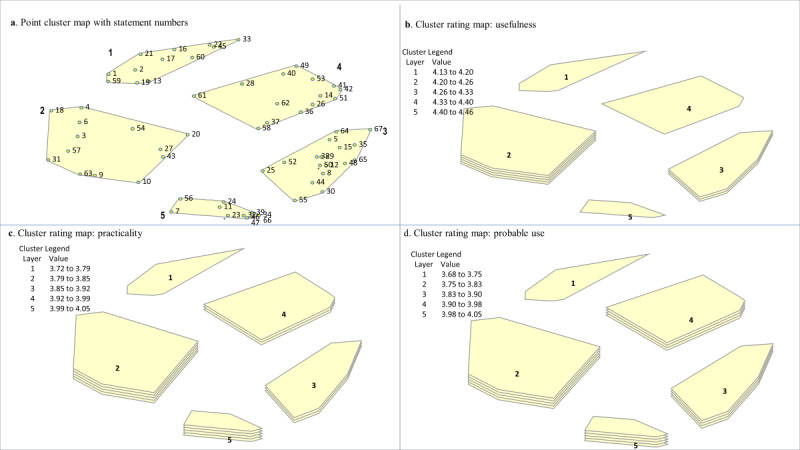
Cluster maps. The five Care Cluster names: (1) Connect (2) Manage (3) Facilitate (4) Provide and (5) Overcome. (a) The point cluster map denotes a 3D nonoverlapping representation of the 5 clusters, determined during the hierarchical cluster analysis process, with their points and statement numbers. Points that are closer together represent statements with a more similar meaning, based on participant sorting. (b) This cluster rating map denotes ratings of the usefulness of each statement, with more layers indicating a cluster with higher average ratings of usefulness for the statements contained within the cluster. Cluster 2 has 5 layers, cluster 3 has 3 layers, and clusters 1, 5, and 4 only have 1 layer. (c) This cluster rating map denotes care staffs’ ratings of the practicality of each statement in their care practice. (d) This cluster rating map denotes care staffs’ probable use ratings in their care practice for a statement.

A total of 93 statements were generated during the brainstorming activity. The researchers consolidated the statements to 67 unique ways that mobile apps could be used to support communication with residents, which were then used in the sorting and rating steps (Multimedia Appendix 1). Data generated during the sorting and rating informed the concept mapping analysis, and all analyses reported hereafter were based on the final cluster map solution. To determine the final cluster solution, a cluster map analysis was performed, which involved generating a range of possible cluster maps (5-10 possible cluster solutions were examined). The larger cluster maps divided clusters into concepts that were deemed to be similar. The research team selected the five-cluster solution, which included concepts that represented nonoverlapping care categories for using mobile technology to support everyday communication. The five-cluster solution had a stress index value of 0.30, which indicated that the cluster map had a good overall fit with the data points and was within the range of most concept mapping projects [43]. Labels for the five-cluster maps were derived from the categories created by participants, and the descriptions were based on the statements included in each cluster, which pertained to the concept overall. The clusters centered around actionable areas of care that occur within the LTC setting. *Care cluster 1: connect* was characterized by 12 statements that focused on using apps to build interpersonal relationships through personalized, meaningful engagement (eg, “Use pictures on the iPad/tablet that are meaningful to the resident [eg, personal history, culture, generational] to stimulate conversation”) and to foster trust and connection during leisure or recreational activities (“Use photos on the iPad/tablet to build trust with residents during recreation activities”). *Care cluster 2: manage* included 13 statements that were around using apps to assess resident needs (eg, health status, behavior, mood, pain, mobility) to provide individualized care (eg, “Use music apps to help residents with their mood and/or emotion”). *Care cluster 3: facilitate* had the highest number of statements (n=17) that focused on using apps to improve staff-resident communication by using verbal and nonverbal forms of communication to meet residents’ individual communication needs (eg, “Use apps with basic sign/symbol functions to communicate with residents”). *Care cluster 4: provide* was categorized by 14 statements that centered around using apps to support residents during the completion of daily tasks and to encourage residents to participate in their self-care (eg, “Use apps with pictures to show residents what care staff will be doing with them during personal care”). Finally, *care cluster 5: overcome* contained 11 statements that pertained to the use of apps to offer a way to reduce or remove cultural-language and/or hearing barriers to engaging residents (eg, “Use apps with speech-to-speech translation function to ‘talk back’ to residents in their language”; Figure 1).

### Statement and Cluster Ratings

The average statement ratings across the 3 variables ranged from 2.89 (“Use apps to communicate with residents in palliative care”) to 4.78 (“Use music apps to help residents with their mood and/or emotion*.*” “Use apps with basic sign/symbol functions to communicate with residents”; Multimedia Appendix 1). No statement was rated, on average, as *somewhat* or *not at all* useful, practical, or likely to use. In terms of perceived usefulness, 2 statements had the highest average rating: “Use music apps to help residents with their mood and/or emotion” and “Use apps with basic sign/symbol functions to communicate with residents” (Multimedia Appendix 1). Care staff rated 3 statements as highest on practicality: “Use pictures on the iPad/tablet that are meaningful to the resident [eg, personal history, culture, generational] to stimulate conversation”; “Use apps that can also translate what care staff say into the language that a resident can understand/speak”; and “Use apps with pictures/text with residents who cannot speak but can point to what they want or need.” Finally*,* 2 statements were rated highest on the care staff’s probable use in their care practice: “Use apps that include both visual and written forms of communication during activity sessions” and “Use apps with pictures/text with residents who cannot speak but can point to what they want or need.”

Overall, the care staff’s average ratings for the 5 care clusters ranged from 4.13 (*care cluster 1: connect*) to 4.46 (*care cluster 2: manage*) on usefulness, from 3.72 (*care cluster 1:* *connect*) to 4.04 (*care cluster 1: overcome*) on practicality, and from 3.68 (*care cluster 1:* *connect)* to 4.03 (*care cluster 1: overcome*) on probable use (Multimedia Appendix 1). The results from the cluster map analyses showed that, relative to other care clusters on the maps, the care staff considered *care cluster 2: manage* to contain statements with the highest ratings for using mobile apps to support everyday communication with residents (Figure 1). The average statement ratings in this care cluster ranged from 3.56 to 4.78 (Multimedia Appendix 1). For example, the statements with the highest average ratings for usefulness were as follows: “Use music apps to help residents with their mood and/or emotion.” (statement number 6: average rating 4.78), “Use apps to ask information about residents’ needs and wants.” (statement number 20: average rating 4.67), and “Use apps to keep an up-to-date record of a resident’s needs.” (statement number 31: average rating 4.67; Multimedia Appendix 1). Care staff ratings indicated that using mobile apps to overcome barriers (*care cluster 5: overcome*) was highly practical and that there was a strong likelihood that they would use mobile apps for this purpose in their care practice. For example, “Using apps that can translate what care staff say into the language that a resident can understand/speak” (statement number 47) was rated, on average, as highly useful (average rating 4.56), practical (average rating 4.56), and likely to use in their care practice (average rating 4.44; Multimedia Appendix 1). Conversely, although *care cluster 1: connect*, on average, was rated as moderate-to-very important in terms of usefulness, practicality, and probable use, it was the care cluster with the lowest average ratings. For example, the statement “Use map apps as a topic of discussion with residents (eg, talk about where they used to live)” was rated lower on both a practical (average rating 3.11) and probable (average rating 3.33) way to support everyday communication in LTC.

### Go-Zone Analysis

**Figure 2:**
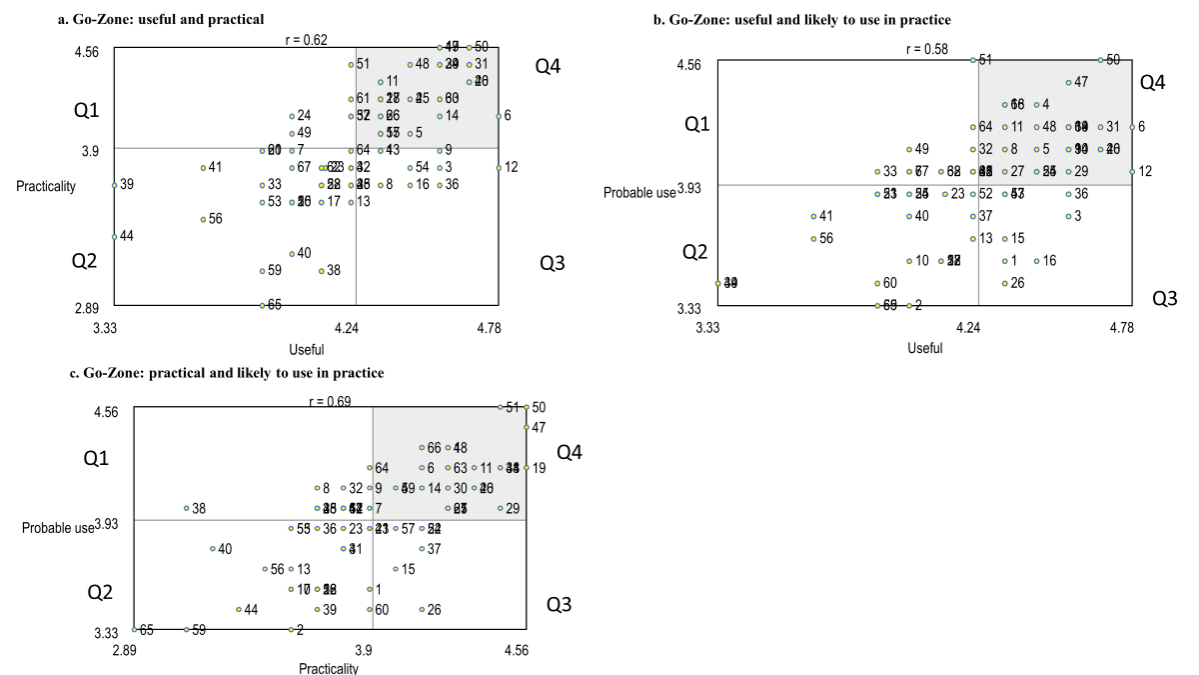
Go-Zone analysis displays comparing statements across the rating criteria. Q4: quadrant 4 (top-right shaded quadrant) of the Go-Zone display represents statements that were rated high on both variables in the comparison (ie, very useful and very likely to use in practice; go-to care zone). Q2: quadrant 2 (bottom-left quadrant) included statements that were deemed lower on both variables (eg, somewhat practical and likely to use). The size and location of the quadrants vary from cluster to cluster because the quadrants are formed by drawing a line at the cluster average of the variable ratings.

The Go-Zone analysis generated 3 visual displays that were derived by comparing care staff’s ratings on the 3 rating variables: (1) usefulness and practicality; (2) usefulness and probable use; and (3) practicality and probable use (Figure 2). Across the 3 comparisons, a total of 20 actionable statements were in quadrant 4, or the *go-to* care zone, meaning that all these statements were rated above average on usefulness, practicality, and probable use (Table 2). The majority (13/20, 65%) of the statements found in the *go-to care zone* were from *care cluster 2: care management* (n=7) and *care cluster 3: facilitate* (n=6). Two care clusters contained only one statement that care staff rated as *very* across all 3 rating comparisons: *connect:* “Use pictures on the iPad/tablet that are meaningful to the resident (personal history, culture, generational) to stimulate conversation.” and *caregiving*: “Use apps with pictograms to help with directions given to residents*.*” Conversely, quadrant 2, or the *no-go care zone* included 18 statements that were commonly rated lower across the 3 variable comparisons, with the majority of the statements (11/18, 61%) included in *care cluster 1: connect* (n=7) and *care cluster 4: provide* (n=4; Table 3). Finally, paired-sample *t* tests were conducted to examine any differences between the overall ratings for the clusters on the different rating variables. There were no statistically significant differences between practicality and probable use for any of the care clusters and no differences in any of the rating variables for cluster 5: *overcome* (all *P*>.05). However, the ratings were statistically significantly different for clusters 1 to 4 on the rating categories of usefulness and probable use (*t* statistic, *P*<.01), indicating that the staff may perceive a statement as useful but less likely to use in the mobile app for this purpose in their care practice.

**Table 2:** The “Go-to” uses of mobile technology to support everyday communication in long-term care across the fourth quadrant of the 3 Go-Zone graphic displays (n=20).

Care cluster and statement number		High-priority statements
**Care cluster 1: connect**		
	19	Use pictures on the iPad/tablet that are meaningful to the resident (eg, personal history, culture, generational) to stimulate conversation
**Care cluster 2: manage**		
	4	Use apps that include a music option for its therapeutic benefits to residents
	6	Use music apps to help residents with their mood and/or emotion
	18	Use art therapy apps with residents who have limited mobility
	20	Use apps to ask information about residents’ needs and wants
	27	Use apps to ask the resident how they are feeling
	31	Use apps to keep an up-to-date record of a resident’s needs
	63	Use apps to assess if the resident is in pain
**Care cluster 3: facilitate**		
	5	Use apps with pictures that residents can use to self-express with care staff
	25	Use translation apps to provide instructions on how to do a task so that residents can understand
	29	Use apps that include pictures, text, and speech to communicate with residents
	30	Use photos on the iPad/tablet to support communication with residents living with hearing loss
	48	Use apps with pictures to communicate with residents
	50	Use apps with pictures/text with residents who cannot speak but can point to what they want or need
**Care cluster 4: provide**		
	14	Use apps with pictograms to help with directions given to residents
**Care cluster 5: overcome**		
	11	Use translation apps with both text-to-text and text-to-speech functions to communicate with residents who do not speak English
	34	Use apps to translate what residents say in other languages into English (eg, speech-to-speech)
	46	Use apps with speech-to-speech translation function to “talk back” to residents in their language
	47	Use apps that can also translate what care staff say into the language that a resident can understand/speak
	66	Use translation apps to help residents who speak other languages to indicate their needs

**Table 3:** Low priority statements identified across the second quadrant of the 3 Go-Zone graph displays (n=18).

Care cluster and statement number		Lower priority ways of using mobile technology to support everyday communication in long-term care
**Care cluster 1: connect**		
	2	Use apps with custom personal videos (eg, family) to connect with residents
	13	Use customizable apps to create communication topics that help staff get to know residents
	17	Use painting apps to communicate with residents
	21	Use photos on the iPad/tablet to build trust with residents during recreation activities
	22	Use apps to engage in fun activities with residents (eg, write stories together)
	59	Use map apps as a topic of discussion with residents (eg, talk about where they used to live)
	60	Use apps to engage in social conversation to get to know residents
**Care cluster 2: manage**		
	10	Use apps that are preprogrammed with a voice that is familiar to residents to help communication
**Care cluster 3: facilitate**		
	44	Use apps to communicate with residents living with dementia
	55	Use translation apps with English-to-English function to help residents understand care staff who have an accent
	65	Use apps to communicate with residents in palliative care
**Care cluster 4: provide**		
	40	Use apps to play simple, short instructional videos of an activity to help communicate with residents
	41	Use apps to inform residents about programs and activities that are happening in the facility
	53	Use apps to invite residents to join programs and activities that are happening in the facility
	58	Use apps with pictures to provide instructions to residents on how to do a task (ie, visual cues)
**Care cluster 5: overcome**		
	23	Use tablets/apps to amplify translated speech for people living with a language barrier and a hearing impairment
	39	Use translation apps with text-to-speech/speech-to-text features to overcome language barriers that residents with Alzheimer disease or dementia face when they no longer speak English
	56	Use tablets/apps to amplify care staff’s speech for people living with a hearing impairment

## Discussion

### Key Ways of Using Mobile Technology to Support Everyday Communication

This study aimed to increase our understanding of the various ways that care staff would use mobile technology to support everyday communication with residents as well as provide insight into which care contexts staff perceive mobile cApps to be most useful, practical, and would likely use with older adults living in LTC homes. The qualitative results of this study identified 67 different ways that mobile apps could be used to support everyday communication between care staff and residents, indicating that care staff recognize a wide range of possible ways of using mobile apps to support communication with residents. Moreover, all the different ways of using mobile apps were rated by staff, on average, as moderately to extremely useful, practical, and would likely use in their care practice. The quantitative results indicated that, generally, the highest-rated ways of using mobile apps with residents were for 3 key purposes: nonpharmacological intervention, AAC, and language translation. Specifically, care staff viewed using music apps to improve residents’ mood or emotion and using mobile apps to provide visual representations (pictures or images and text) that support communication with residents as most useful. Furthermore, care staff indicated that using mobile apps to present meaningful pictures to stimulate conversation, using apps with a translation feature, and using apps with pictures or text to help people who no longer speak to be most practical. Finally, care staff indicated that they would most likely use mobile apps during activities that include both visual and written forms of communication to help support people who have limited verbal communication.

The participants’ emphasis on using music to manage the care needs of residents aligns with evidence that music can play an important role in communication [48,49]. The enjoyment of music involves sensory, cognitive (attention, memory, and language), and emotional processing, with the pleasure of music offering a therapeutic approach for individuals living with mood disturbances [50]. People living with dementia continue to enjoy the mood benefits of music and respond to music even in later stages of the disease when verbal forms of communication are limited [51]. Indeed, there is now a growing body of evidence reporting the benefits of music therapy based on a reduction in disruptive behaviors, anxiety, and depressive moods [52-55]. Given the reported benefits of music in the literature, it is not surprising that care staff identified the therapeutic use of adding music into their care practice toolbox.

Care staff perceived using mobile apps that offer multiple communication modes (pictures or images, text, and speech) to meet residents’ needs or preferences as useful, practical, and likely to be used in their care practice. AAC tools and techniques supplement or replace speech for those living with a communication disorder that impacts language production and/or comprehension [56]. AAC tools aim to engage, connect, and improve the quality of life of people living with spoken and/or written communication impairments resulting from dementia and their care partners [57]. There has been a wide range of traditional nontech (eg, gestures, signing, facial expression, body language, vocalizations) and low-tech AAC options (eg, picture communication boards or books, memory books, communication or memory wallet, photos, objects, paper and pen for written messages, written choice cards, printed reminders) available to support the complex communication needs of people living with dementia so that they can express their wants or preferences as well as connect with their care partners [56-63].

With recent advances in mainstream technologies (eg, tablets or smartphones and their apps), there is a growing number of high-tech AAC solutions that have been developed to address a range of communication needs for people living with communication disorders [57,64]. For example, several traditional low-tech AAC tools have been adapted to high-tech formats (eg, digital communication books, digital memory books, mobile reminiscence, multimedia videos, electronic picture boards) [57,65-72]. AAC tools available as mobile apps (eg, *GoTalk NOW*) offer several potential advantages over low-tech AAC solutions in the LTC setting, including ease of access, portability, size and storage, variety of features, low cost, and range of customization. Furthermore, the key advantages of using mobile AAC apps with residents are that they can include multiple communication modes (pictures or images, music, text, and speech) and can be personalized to support residents’ individual communication needs and preferences [60,73]. Although mobile AAC apps have the exciting potential to offer LTC staff and residents innovative communication solutions that can be adapted to the users’ ability level, there is a need to develop and evaluate evidence-informed mobile apps that aim to address the communication needs of people living with dementia and their caregivers generally [74] and specific to the LTC home setting [75].

Canada is a culturally and linguistically diverse country, with more than 1 in 5 Canadians being foreign-born citizens [76]. Health care settings located in major urban areas (eg, Vancouver, Toronto, Montreal) comprise cultural-linguistically diverse staff and patients, making language barriers a common issue that can impact equitable assessment of care, treatment, health outcomes, quality of care, and patient satisfaction [77,78]. Estabrooks et al [79] surveyed care aides working in LTC homes across western Canada to better understand demographics. Findings from their study highlighted that the majority of care aide respondents were not born in Canada and that English was not their first language [79]. Although the use of professional medical interpretive services (eg, telephone, video, in person) is the standard practice in a health care setting to connect residents and care providers, these resources are limited in terms of cost, access, and time and are not always available on demand to support everyday communication taking place during daily care routines [13,80,81]. Access limitations mean that residents’ immediate needs may go unmet, leading to frustrations for both residents and care staff. Although learning some basic words in the resident’s language or requesting assistance from a coworker who speaks the resident’s language may offer an occasional solution [6], there is a need for care staff to have access to on-demand translation tools available in the mobile app marketplace. It has been recognized that using commercially available mobile translation apps in the health care setting has raised concerns about the risks of inaccurate translations of important health care information, with some studies indicating poor translation accuracy of medical information or phrases in popular apps such as *Google Translate* [82-84]. However, although accuracy is a major concern for communicating sensitive medical information (diagnosis, treatment, and consent), using mobile apps for everyday communication during daily activities (eg, identifying pain, toileting, dressing, and mealtime) may be of less risk. Indeed, a recent study by Panayiotou et al [81] identified 15 free commercially available translation apps in the Apple iTunes Store and evaluated their suitability for everyday communication with older adults in health care settings. The results indicated that 2 translation apps designed for the health care setting, *CALD Assist* and *Talk To Me*, were most suitable for translating everyday communication, as the apps were limited to preset phrases that could be used during noncritical care contexts (eg, communicating care needs) [81]. As is the case with AAC apps, although there are a few apps showing promise for supporting translation during everyday communication in health care settings, caution needs to be taken when using unregulated, commercially available translation apps that have not been examined clinically or empirically for use in LTC [13].

### Key Care Contexts for Using Mobile Technology

Care staff categorized the different ways of using mobile apps to support everyday communication into 5 key care contexts, such as building interpersonal relationships through shared activities and encouraging residents’ participation in their self-care tasks. Across all identified care contexts, providing individualized care appeared to be the purpose of using mobile apps to support everyday communication with residents living in LTC homes. The cornerstone of person-centered care in health care settings is the provision of individualized care by gathering information about an individual’s values, needs, personal history, and preferences to better understand their health care goals as well as encouraging participation in one’s own health care decisions [85-90]. Person-centered approaches to care have been developed to address the needs of people living in LTC homes [91-95]. Person-centered dementia care was founded on the principle of personhood and emphasized the importance of relationships in the LTC context [92,96,97]. Person-centered dementia care has evolved into a care model that includes 4 fundamental elements: (1) value people living with dementia and their caregivers; (2) treat people living with dementia as individuals with unique needs and preferences; (3) consider the perspectives of people living with dementia to help better understand their reality; and (4) create a positive social environment that supports communication, fosters interpersonal relationships, and promotes well-being [98-100]. The care contexts identified in this study overlap with several key principles of person-centered dementia care within the LTC home setting, including effective communication (*care clusters:* *facilitate and overcome*), individualized care (*care clusters: manage and provide*), and building social relationships and engaging in meaningful activities (*care cluster*: *connect*). Furthermore, this study’s findings align with components of the recently published person-centered dementia care practice recommendations: (1) know the person living with dementia (need, preference, history, values, beliefs, interest, and abilities) to inform everyday encounters; (2) recognize and accept the person’s reality and know that behavior is communication; (3) identify and support ongoing opportunities for meaningful engagement that support interests and preferences; and (4) build and nurture authentic caring relationships that focus on the relationship and not only the task [101,102].

This study’s Go-Zone analysis highlighted the ways of using mobile apps that are high priority (ie, rated *very* to *extremely*) and the care contexts that care staff considered most useful, practical, and likely to use in their care practice to support everyday communication with residents (ie, *go-to care zones*). These findings offer a better understanding of where to target communication interventions that use mobile apps. Specifically, care staff perceived 3 care contexts to be most useful, practical, and likely to use mobile apps with residents to support communication: *care management, facilitating communication, and overcoming barriers*. The context of managing care was characterized by activities that focused on assessing and/or responding to residents’ care needs. In addition to including several ways of using music apps for therapeutic purposes, this care context focused on using mobile apps to identify residents’ needs and wants, to keep up-to-date records of these needs, and to assess pain. Chronic pain is a common symptom among older adults living in LTC homes [103-105] resulting from comorbid conditions (eg, injury, surgery, and disease) [106]. Given the prevalence of pain in the LTC home setting and that pain assessment and management are further challenged for residents living with dementia, recognizing and treating residents’ pain needs improvement. Indeed, it is not surprising that care staff identified pain assessment as a priority in the LTC setting, as using innovative tools to better detect and effectively treat pain among residents living in LTC homes [107] would help to improve the well-being and quality of life of residents living with pain [105,108].

Care staff perceived the majority statements included in the *care cluster:* *connect* as moderately important for supporting everyday communication with residents during daily activities*. Care cluster: connect* included a dozen statements that focused on using mobile apps to foster positive caregiver-resident interpersonal relationships through shared activities and meaningful engagement. Interestingly, although care staff perceive several ways of using mobile apps to be useful for supporting social participation and nurturing relationships, care staff may have experienced or, possibly foreseen, challenges with implementing mobile technology interventions for this purpose in their care practice. For example, while *centered* approaches to resident care are beneficial [93], building and nurturing resident–care staff relationships takes time, staffing resources, and care staff education or training. Staffing and environment constraints, high workload demands, time pressure, workplace culture, limited experience, and/or training can hinder *centered* approaches to care [109-111]. Indeed, the *care cluster: connect* encompasses the principles of relationship-centered care, which shifts the focus of care beyond the individual (ie, person-centered care) to include the relational and social contexts of care [89,112,113]. In the relationship-centered care approach, more emphasis is placed on relationships, including the resident–care staff relationship. This approach focuses on enhancing the dyad’s care experience and cultivating a reciprocal relationship that meets both the residents’ and the care staff’s needs. Building and nurturing relationships take time and would require changes to the focus of care practice approaches, with greater emphasis placed on relationship-oriented care over task-oriented care.

### Strengths and Limitations

To our knowledge, this is the first study to explore care staff’s perspectives about using mobile technology to support everyday communication with older adults living in LTC homes during daily activities. This study demonstrated the feasibility of using the mixed methods concept mapping approach with care staff in the LTC setting to identify the various ways that mobile apps could be used with residents to support communication and highlight priority care contexts to target future mHealth interventions. Employing the concept mapping approach offered a way to promote care staff engagement in the research process as well as capitalize on group discussion to quickly generate various ideas that may not be captured during in-depth interviews. In addition, this efficient and timely method offered flexibility in the research process. However, the concept mapping method is limited in its ability to explore care staff’s perspectives in greater detail. Therefore, to enhance concept mapping results, future research should consider including traditional interviews concurrent with the concept mapping method or following up on key findings with interviews to capture both the breadth and the depth around care staff perspectives on using mHealth in LTC. Indeed, the combination of concept mapping methods and focus group interviews has been shown to produce complementary results, capturing the complexities of a topic under inquiry [34].

Although methods used in concept mapping are suitable for any sample size above 10 [43], the small sample size in this study means that the findings cannot be generalized to other care staff in the LTC setting. Furthermore, this study included only one male; thus, this study may overlook the unique perspectives of male care staff. Participating care staff did not engage in the interpretation phase of this study (cluster map name and description consensus). Therefore, participant engagement was limited to 2 phases of the study, and interpretations were based on the research team consensus. In addition, although the information gathered from care staff is the first step to better understand the ways mobile technology could be used with residents, this study acknowledges that resident perspectives were not included. Future research should consider including residents in the research process to better assist in identifying key areas that they would want to use technology with care staff.

### Conclusions

Effective communication is fundamental to the provision of person-centered care. According to care staff, there are a variety of ways to use mobile apps to support communication with residents living in LTC homes. Care staff categorized the various ways of using mobile apps with residents into 5 care contexts. The findings expand our understanding of priority areas for using mobile apps with residents in LTC homes, which included using mobile apps to support communication during care management activities, to facilitate verbal and nonverbal communication to meet residents’ individual needs, and to overcome cultural-language barriers. This study demonstrated that concept mapping is a useful tool for engaging caregivers in the research process to illuminate caregivers’ perspectives around using mobile apps to support communication with older adults living in LTC homes. Using mobile apps to deliver interventions (eg, AAC and nonpharmacological) is a key area for future research and clinical practice. For example, using a mobile app to measure health status could be employed as part of a resident’s care plan to support person-centered care or using a mobile app to assess pain offers residents a way to communicate their care needs. This study provides an initial understanding of the ways in which mobile apps could be used to support caregiver-resident communication. Identifying priority care areas for using mobile apps is essential for targeting innovative mHealth interventions designed to support and enhance resident-caregiver communication in the LTC setting, ultimately improving person-centered care and residents’ quality of life.

## Appendix

Multimedia Appendix 1 Conceptual clusters: generated statements and average ratings on usefulness, practicality, and probability of use.

## Abbreviations

AAC: augmentative and alternative communication
ADL: activity of daily living
cApps: communication applications
LTC: long-term care
mHealth: mobile health
