# Supplementary material for: Care Staff Perspectives on Using Mobile Technology to Support Communication in Long-Term Care: Mixed Methods Study
Source: JMIR Nurs. 2020 Sep 29;3(1):e21881. doi: 10.2196/21881 (PMC8373373; doi:10.2196/21881)
Supplement: Multimedia Appendix 1 [file nursing_v3i1e21881_app1.docx]

**Multimedia Appendix Table 1.** Conceptual clusters: generated statements and average ratings on usefulness, practicality, and probability of use

| Item number | Cluster statements | Ratings**^a^** | | | | |
| --- | --- | --- | --- | --- | --- | --- |
|  |  | Bridging^b^ | Usefulness | Practicality | Probable use |  |
|  | | | | | | |
| **Care cluster 1: connect (n=12), mean (SD)** | | 0.23 (0.08) | 4.13 (0.22) | 3.72 (0.32) | 3.68 (0.27) |  |
| 1 | Use an iPad/tablet with photos of a resident’s personal history to connect with them during recreation activities | 0.24 | 4.33 | 3.89 | 3.56 |  |
| 2 | Use apps with custom personal videos (eg, family) to connect with residents | 0.17 | 4 | 3.56 | 3.33 |  |
| 13 | Use customizable apps to create communication topics that help staff get to know residents | 0.37 | 4.22 | 3.56 | 3.67 |  |
| 16 | Use apps that include hobbies (eg, painting, cross-words) so that care staff could connect with residents while they engage in activities | 0.19 | 4.44 | 3.67 | 3.56 |  |
| 17 | Use painting apps to communicate with residents | 0.28 | 4.11 | 3.56 | 3.56 |  |
| 19 | Use pictures on the iPad/tablet that are meaningful to the resident (eg, personal history, culture, generational) to stimulate conversation | 0.38 | 4.56^c^ | 4.56^c^ | 4.22 |  |
| 21 | Use photos on the iPad/tablet to build trust with residents during recreation activities | 0.1 | 3.89 | 3.89 | 3.89 |  |
| 22 | Use apps to engage in fun activities with residents (eg, write stories together) | 0.19 | 4.11 | 3.67 | 3.56 |  |
| 33 | Use cognitive games to engage residents (independent or with care staff) | 0.19 | 3.89 | 3.67 | 4 |  |
| 45 | Use apps with games to connect with residents | 0.16 | 4.22 | 3.67 | 4 |  |
| 59 | Use map apps as a topic of discussion with residents (eg, talk about where they used to live) | 0.29 | 3.89 | 3.11 | 3.33 |  |
| 60 | Use apps to engage in social conversation to get to know residents | 0.24 | 3.89 | 3.89 | 3.44 |  |
| **Care cluster 2: manage (n=13), mean (SD)** | | 0.55 (0.17) | 4.46 (0.19) | 4.05 (0.25) | 4.05 (0.22) |  |
| 3 | Use apps to help change the resident’s mood when he/she refuses care | 0.59 | 4.56^c^ | 3.78 | 3.78 |  |
| 4 | Use apps that include a music option for its therapeutic benefits to residents | 0.45 | 4.44 | 4.22 | 4.33 |  |
| 6 | Use music apps to help residents with their mood and/or emotion | 0.42 | 4.78^c^ | 4.11 | 4.22 |  |
| 9 | Use music (eg, apps or saved on iPad/tablet) to ease/calm communication | 0.58 | 4.56^c^ | 3.89 | 4.11 |  |
| 10 | Use apps that are preprogrammed with a voice that is familiar to residents to help communication | 0.59 | 4 | 3.56 | 3.56 |  |
| 18 | Use art therapy apps with residents who have limited mobility | 0.69 | 4.33 | 4.22 | 4.33 |  |
| 20 | Use apps to ask information about residents’ needs and wants | 0.37 | 4.67^c^ | 4.33 | 4.11 |  |
| 27 | Use apps to ask the resident how they are feeling | 0.42 | 4.33 | 4.22 | 4 |  |
| 31 | Use apps to keep an up-to-date record of a resident’s needs | 1 | 4.67^c^ | 4.44 | 4.22 |  |
| 43 | Use apps to ask a resident for information about their health/illness | 0.44 | 4.33 | 3.89 | 3.89 |  |
| 54 | Use music apps to connect with residents (ie, music as a form of communication) | 0.49 | 4.44 | 3.78 | 4 |  |
| 57 | Use music apps to help residents with their behavior | 0.44 | 4.33 | 4 | 3.89 |  |
| 63 | Use apps to assess if the resident is in pain | 0.69 | 4.56^c^ | 4.22 | 4.22 |  |
| **Care cluster 3: facilitate (n=17), mean (SD)** | | 0.21 (0.03) | 4.27 (0.33) | 3.86 (0.45) | 3.97 (0.28) |  |
| 5 | Use apps with pictures that residents can use to self-express with care staff | 0.2 | 4.44 | 4 | 4.11 |  |
| 8 | Use apps with pictograms/pictographs to support nonverbal communication with residents | 0.2 | 4.33 | 3.67 | 4.11 |  |
| 12 | Use apps with basic sign/symbol functions to communicate with residents | 0.16 | 4.78^c^ | 3.78 | 4 |  |
| 15 | Use apps to support discussions with residents who rely on nonverbal communication during therapeutic work | 0.23 | 4.33 | 4 | 3.67 |  |
| 25 | Use translation apps to provide instructions on how to do a task so that residents can understand | 0.21 | 4.44 | 4.22 | 4 |  |
| 29 | Use apps that include pictures, text, and speech to communicate with residents | 0.19 | 4.56^c^ | 4.44 | 4 |  |
| 30 | Use photos on the iPad/tablet to support communication with residents living with hearing loss | 0.23 | 4.56^c^ | 4.22 | 4.11 |  |
| 35 | Use apps with a writing option to help communicate with residents | 0.24 | 4.22 | 3.67 | 4 |  |
| 38 | Use writing apps (eg, use a finger or stylus pen) that support communication between residents and care staff | 0.18 | 4.11 | 3.11 | 4 |  |
| 44 | Use apps to communicate with residents living with dementia | 0.21 | 3.33 | 3.33 | 3.44 |  |
| 48 | Use apps with pictures to communicate with residents | 0.2 | 4.44 | 4.44 | 4.22 |  |
| 50 | Use apps with pictures/text with residents who cannot speak but can point to what they want or need | 0.16 | 4.67^c^ | 4.56^c^ | 4.56 |  |
| 52 | Use apps to communicate with any resident who cannot communicate in “traditional” ways | 0.25 | 4.22 | 4.11 | 3.89 |  |
| 55 | Use translation apps with English-to-English function to help residents understand care staff who have an accent | 0.18 | 4 | 3.56 | 3.89 |  |
| 64 | Use an iPad/tablet to help residents understand care staff | 0.26 | 4.22 | 3.89 | 4.22 |  |
| 65 | Use apps to communicate with residents in palliative care | 0.21 | 3.89 | 2.89 | 3.33 |  |
| 67 | Use apps that can help to verify that residents understand what the care staff have said to them | 0.28 | 4 | 3.78 | 4 |  |
| **Care cluster 4: provide (n=14), mean (SD)** | | 0.27 (0.08) | 4.17 (0.23) | 3.87 (0.31) | 3.92 (0.26) |  |
| 14 | Use apps with pictograms to help with directions given to residents | 0.2 | 4.56^c^ | 4.11 | 4.11 |  |
| 26 | Use apps to ask if the resident wants to use the toilet | 0.21 | 4.33 | 4.11 | 3.44 |  |
| 28 | Use art therapy apps to communicate with residents | 0.38 | 4.22 | 3.67 | 4 |  |
| 36 | Use apps with pictures to show residents what care staff will be doing with them during personal care | 0.17 | 4.56^c^ | 3.67 | 3.89 |  |
| 37 | Use apps that allow residents to request assistance from care staff | 0.18 | 4.22 | 4.11 | 3.78 |  |
| 40 | Use apps to play simple, short instructional videos of an activity to help communicate with residents | 0.33 | 4 | 3.22 | 3.78 |  |
| 41 | Use apps to inform residents about programs and activities that are happening in the facility | 0.29 | 3.67 | 3.78 | 3.78 |  |
| 42 | Use apps to communicate the schedule of daily activities with residents | 0.28 | 4.22 | 3.78 | 4 |  |
| 49 | Use apps to ask residents what activities they would like to do | 0.28 | 4 | 4 | 4.11 |  |
| 51 | Use apps that include both visual and written forms of communication during activity sessions | 0.42 | 4.22 | 4.44 | 4.56^c^ |  |
| 53 | Use apps to invite residents to join programs and activities that are happening in the facility | 0.3 | 3.89 | 3.56 | 3.89 |  |
| 58 | Use apps with pictures to provide instructions to residents on how to do a task (ie, visual cues) | 0.22 | 4.11 | 3.67 | 3.56 |  |
| 61 | Use apps that can include personal photos to communicate with residents | 0.38 | 4.22 | 4.22 | 4 |  |
| 62 | Use apps that help residents to choose what food they want to eat during mealtime | 0.2 | 4.11 | 3.78 | 4 |  |
| **Care cluster 5: overcome (n=11), mean (SD)** | | 0.14 (0.18) | 4.16 (0.38) | 4.04 (0.34) | 4.03 (0.28) |  |
| 7 | Use apps with a sign language function to support translation for people who use sign language | 0.42 | 4 | 3.89 | 4 |  |
| 11 | Use translation apps with text-to-text and text-to-speech functions to communicate with residents (non-English speaking) | 0.22 | 4.33 | 4.33 | 4.22 |  |
| 23 | Use tablets/apps to amplify translated speech for people living with a language barrier and a hearing impairment | 0.08 | 4.13 | 3.78 | 3.89 |  |
| 24 | Use translation apps to let residents know what staff are doing with them during personal/ hygiene care | 0.21 | 4 | 4.11 | 3.89 |  |
| 32 | Use translation apps to communicate with a resident’s relatives or visitors in their language | 0.02 | 4.22 | 3.78 | 4.11 |  |
| 34 | Use apps to translate what residents say in other languages into English (eg, speech-to-speech) | 0.02 | 4.56^c^ | 4.44 | 4.22 |  |
| 39 | Use translation apps with text-to-speech/speech-to-text features to overcome language barriers that residents with Alzheimer disease or dementia face when they no longer speak English | 0.07 | 3.33 | 3.67 | 3.44 |  |
| 46 | Use apps with speech-to-speech translation function to “talk back” to residents in their language | 0 | 4.67^c^ | 4.33 | 4.11 |  |
| 47 | Use apps that can also translate what care staff say into the language that a resident can understand/speak | 0 | 4.56^c^ | 4.56^c^ | 4.44 |  |
| 56 | Use tablets/apps to amplify care staff’s speech for people living with a hearing impairment | 0.5 | 3.67 | 3.44 | 3.67 |  |
| 66 | Use translation apps to help residents who speak other languages to indicate their needs | 0.02 | 4.33 | 4.11 | 4.33 |  |

^a^Average ratings were based on a Likert type scale: 1=not at all, 2=somewhat, 3=moderately, 4=very, and 5=extremely

^b^ Concept System Global MAX uses built-in proprietary indexes for calculating a bridging value for any statement or cluster from the combination of the original sort data and the multidimension scaling results. Numerical values are calculated to assist with interpretation. Average bridging values range from 0 to 1 and indicate the degree to which an item was sorted with other items in the same cluster (value closer to 0) or sorted with items belonging to another cluster (value closer to 1). Care clusters 1, 3, 4, and 5 had lower bridging values (0.14-0.21), indicating statements with a closer relationship. Care cluster 2 demonstrated less agreement than the other clusters on how the statements were sorted (bridging value=0.55), with statements 18 and 63 having the highest bridging value (0.69).

**^c^**The highest-rated statements (4.5 or higher) for each rating variable.
